# HUSH, NEXT PROMPT: Epigenetics and the Nuclear RNA Exosome in Human Aging and Disease

**DOI:** 10.3390/cells15151378

**Published:** 2026-07-30

**Authors:** Andrew G. Newman, Prim B. Singh

**Affiliations:** 1 Institute of Cell and Neurobiology, Charité—Universitätsmedizin Berlin, Corporate Member of Freie Universität Berlin and Humboldt-Universität zu Berlin, 10117 Berlin, Germany; 2 Department of Biosciences, School of Medicine, Nazarbayev University, 5/1 Kerei, Zhanibek Khandar Street, Astana 010000, Kazakhstan; prim.singh@nu.edu.kz

**Keywords:** RNA exosome, transposable elements, epigenetics

## Abstract

The nuclear RNA exosome, a conserved 3′→5′ ribonuclease complex, degrades the vast majority of RNA polymerase II output, including promoter upstream transcripts, enhancer RNAs, antisense transcripts, and retrotransposon-derived RNAs. Beyond this housekeeping role, the exosome acts as an epigenetic effector, and its dysfunction underlies a growing spectrum of human disease. Here we integrate recent structural, genomic, and disease-focused studies into a unified model of the exosome as a guardian of the epigenome. We describe how MTR4-containing adaptor complexes TRAMP, NEXT, and PAXT confer substrate selectivity, and how the exosome enforces heterochromatic silencing in concert with HP1 proteins and the Human Silencing Hub (HUSH) complex and preserves three-dimensional genome architecture at insulators and enhancers, such as the protocadherin locus where RNA surveillance, CTCF insulation, and heterochromatin converge. We then examine the consequences of failure: exosomopathies such as pontocerebellar hypoplasia, loss of DIS3- and PAXT-mediated tumor suppression in cancer, and age-related erosion of surveillance that permits transposable element de-repression, RIG-1/MDA5 and cGAS-STING-driven inflammation, cellular senescence, and neurodegeneration. We conclude that the exosome couples RNA decay to epigenetic state across the lifespan, positioning RNA surveillance as an emerging therapeutic target.

## 1. Introduction

RNA polymerase II transcribes the vast majority of the mammalian genome, yet most of this output, including promoter upstream transcripts (PROMPTs), enhancer RNAs (eRNAs), antisense transcripts, and retrotransposon-derived RNAs, must be rapidly degraded [1]. The machinery principally responsible for this turnover is the nuclear RNA exosome, a conserved multi-subunit ribonuclease complex first identified in *Saccharomyces cerevisiae* [2]. Beyond its housekeeping role, the exosome also functions as an epigenetic effector: it enforces transcriptional silencing at heterochromatin by degrading noncoding RNAs that would otherwise accumulate [3,4], shapes Polycomb Repressive Complex 2 (PRC2) activity by clearing poly(A)+ transcripts that titrate PRC2 away from chromatin [5], influences three-dimensional genome architecture by processing RNAs at insulator elements and enhancers [6,7], and collaborates with the Human Silencing Hub (HUSH) complex and HP1 proteins to degrade retrotransposon transcripts that trigger innate immune activation [8,9,10,11].

Critically, the noncoding transcripts subject to this surveillance are not merely transcriptional noise, and the exosome does not act on them indiscriminately. Because the exosome degrades the bulk of Pol II noncoding output, a transcript’s stability is determined largely by whether it can evade decay. Conserved, functional long noncoding RNAs (lncRNAs) are enriched among the escapers: they are either retained in chromatin-bound ribonucleoprotein complexes or exhibit stabilizing features such as splicing or protective 3′-end structures in place of a poly(A) tail, such as the triple helices in lncRNAs MALAT1 and NEAT1 that block 3′→5′ exonucleolytic attack [12]. Although such lncRNAs typically show poor primary-sequence conservation, their functions are conserved across evolutionarily distant species through selection on secondary structure, genomic synteny, and conserved protein partners [13,14,15].

In addition to RNA degradation the exosome is also important for processing noncoding RNA: through distributive 3′-end trimming it matures a conserved repertoire of structured ncRNAs, including ribosomal, small nucleolar, and small nuclear RNAs [16,17]. The exosome thus functions as a curator that sets the threshold separating functional noncoding RNAs from pervasive transcriptional noise—a regulated surveillance program rather than a disposal system.

When this surveillance system fails, the consequences are severe. Hypomorphic mutations in exosome subunits cause pontocerebellar hypoplasia and other neurodevelopmental syndromes (reviewed in [18]). Recurrent mutations in DIS3, a catalytic subunit of exosomes, are found in multiple myeloma [19,20,21]. Furthermore, acute exosome depletion triggers nuclear RNA aggregation and premature senescence [22]. The nuclear RNA exosome is therefore a guardian of the epigenome whose dysfunction underlies several developmental diseases, cancer, and age-related diseases such as neurodegeneration.

This review integrates recent findings to present a cohesive model of the nuclear RNA exosome as an epigenetic regulatory complex. We examine its architecture and adaptor complexes (Section 2 and Section 3), chromatin-level functions including the HP1-RNA-exosome axis and the HUSH complex (Section 4), the protocadherin gene cluster as a case study in heterochromatin-dependent gene choice (Section 5), exosomopathies (Section 6), cancer (Section 7), the role of exosome decline in transposable element de-repression, senescence, and aging (Section 8), and pharmacological interventions and future directions (Section 9 and Section 10). For additional comprehensive reviews of exosome biology, see Ogami and Suzuki (2021) [23] and Rambout and Maquat (2024) [24].

This article is an expert narrative review rather than a systematic review or meta-analysis. References were selected by beginning from a core set of foundational primary studies and authoritative reviews on the nuclear RNA exosome and expanding these through backward and forward citation tracing, supplemented by targeted PubMed and Google Scholar searches of the specific sub-topics addressed in each section. We prioritized primary research articles and gave particular weight to recent, high-impact, and mechanistically definitive work, citing comprehensive reviews where a full primary treatment was beyond the scope of this article.

## 2. Architecture and Catalytic Mechanism

**Figure 1:**
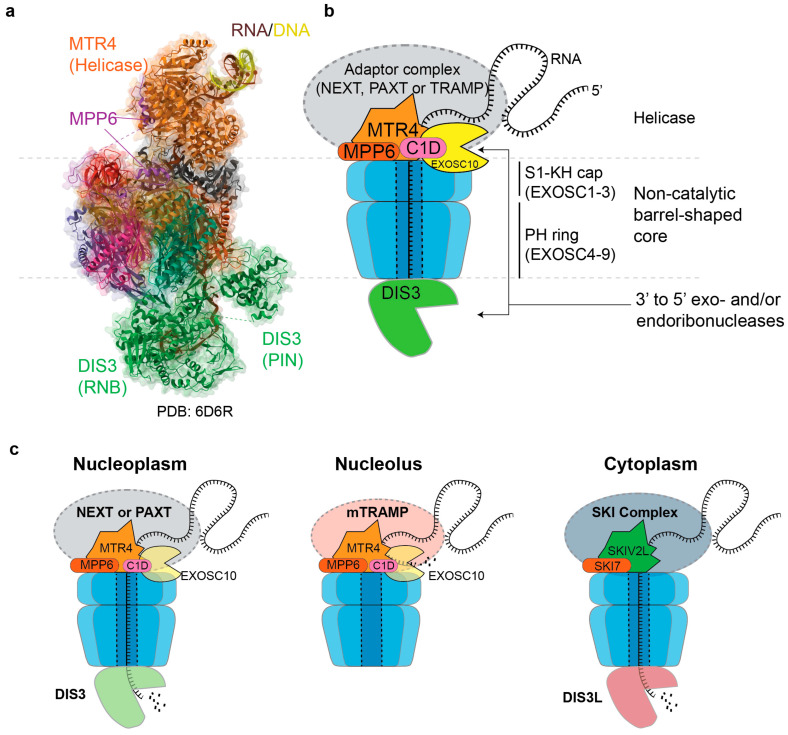
Architecture and compartment-specific configurations of the mammalian RNA exosome. (**a**) Cryo-EM structure of the human nuclear exosome holoenzyme (PDB: 6D6R; [26]), with the MPP6-bound configuration independently determined [27]: the MTR4 helicase (orange) is positioned above the barrel-shaped core, the cofactor MPP6 (purple), and the catalytic subunit DIS3 with its separate ribonuclease (RNB) and endonuclease (PIN) domains (green). Substrate RNA (and a DNA strand) is shown threading through the complex (RNA, brown; DNA, yellow). (**b**) Schematic of the holoenzyme. A single MTR4-containing adaptor complex (NEXT, PAXT or mTRAMP) docks onto the catalytically inert, barrel-shaped nine-subunit core (S1/KH cap, EXOSC1-3; PH-like ring, EXOSC4-9). Reconstitution and structural analysis of the yeast and human cores established that the ring is inert and threads single-stranded RNA 3′→5′ through the central channel to DIS3 (exo- and endonuclease) at the base [25,28,29], while the distributive exonuclease RRP6/EXOSC10, anchored by C1D/RRP47, acts at the cap, with substrates partitioning between the two active sites according to RNA structure [30,31]. (**c**) Compartment-specific configurations. In the nucleoplasm, the core associates with the adaptor complexes NEXT or PAXT through MTR4 and MPP6 and is served by both catalytic nucleases, DIS3 and EXOSC10. In the nucleolus, the mTRAMP complex (scaffold ZCCHC7 with PAPD5/PAPD7) directs the exosome to rRNA precursors where degradation is dominated by EXOSC10, as DIS3 is largely excluded from this compartment [32,33]. In the cytoplasm, the core instead partners with the SKI complex, in which the helicase SKIV2L replaces MTR4 and the adaptor SKI7 (in place of MPP6) bridges the SKI complex to the core; the cytoplasmic-specific catalytic subunit DIS3L replaces DIS3. Across all compartments the nine-subunit core is invariant; specificity is conferred by the associated helicase, adaptor and catalytic subunit.

The RNA exosome is among the most deeply conserved machineries of eukaryotic RNA metabolism; it was discovered in *S. cerevisiae* as a complex of 3′-to-5′ exoribonucleases required for 5.8S rRNA processing [2]. Structural studies revealed that the eukaryotic core is a nine-subunit (Exo-9) assembly: a hexameric barrel of RNase PH-like proteins (EXOSC4-9) capped by a trimer of S1/KH domain proteins (EXOSC1-3) [25] (Figure 1).

Although the barrel subunits are derived from phosphorolytic RNase PH enzymes, the eukaryotic barrel is catalytically inert and instead channels single-stranded RNA toward distally positioned catalytic subunits [28]. Catalytic activity resides in two structurally distinct ribonucleases. DIS3 (Rrp44), a processive hydrolytic exoribonuclease with an additional endonucleolytic PIN domain, is positioned at the barrel base and receives RNA threaded through the central channel [28,29]. EXOSC10 (Rrp6) is a distributive hydrolytic exoribonuclease that lacks endonuclease activity and is anchored at the S1/KH cap near the channel entrance, stabilized by its partner C1D/RRP47 [30]. Routing of substrates to either active site depends on RNA structure: unstructured RNAs thread to DIS3, whereas structured or stalled RNAs are diverted to EXOSC10 [31]. Cryo-EM of the human nuclear exosome confirmed conservation of this dual-path architecture, revealing a longer RNA channel (39–43 nucleotides versus 35–39 in yeast) [27].

The exosome’s architecture is best understood as an elaboration of an ancient phosphorolytic exoribonuclease. The exosome’s ring-forming subunits belong to the RNase PH superfamily, the fold that also builds two ring-shaped bacterial exoribonucleases, the trimeric polynucleotide phosphorylase (PNPase) and the hexameric RNase PH. Because this same fold builds the exosome cores of archaea and eukaryotes as well, a ring-shaped 3′→5′ exoribonuclease predates the divergence of the three domains of life [34,35]. This ancestral state is preserved in the archaeal exosome, a hexameric Rrp41-Rrp42 ring capped by S1/KH proteins with catalytic sites at the subunit interfaces; like PNPase, it degrades RNA 3′→5′ by phosphorolysis, a phosphate-dependent and reversible reaction [36,37]. The eukaryotic exosome arose through two coupled changes—the six ring subunits (EXOSC4-9) lost their catalytic residues to become a purely structural channel, and catalysis was outsourced to recruited hydrolytic nucleases (DIS3 and EXOSC10). This shift from phosphorolytic to hydrolytic chemistry, and from ring-intrinsic to recruited catalysis, rendered degradation effectively irreversible and allowed a single conserved core to be directed toward either processive destruction or precise 3′-end processing [17,29,38]. The invariant Exo-9-DIS3 core is conserved from yeast to humans, whereas the nuclear-specific elaborations EXOSC10, the MTR4/MTREX helicase, and the TRAMP, NEXT and PAXT adaptors, along with expansion of the DIS3 paralogs (DIS3, DIS3L, DIS3L2), reflect progressive specialization of substrate recruitment [17,32].

At the barrel top, MTR4 (mRNA transport 4; gene name MTREX) serves as the universal entry point for nuclear substrates. MPP6 tethers MTR4 to the cap, while C1D (Rrp47) stabilizes EXOSC10 [26,30]. RNA-engaged MTR4 displaces the catalytic module of EXOSC10, suggesting the two RNA access routes are mutually exclusive [26]. MTR4’s role as gatekeeper ensures that every substrate must first be unwound and presented at the channel mouth, providing a critical checkpoint for adaptor-mediated regulation. MTR4 is a Ski2-like DExH-box helicase, and its cytoplasmic counterpart SKIV2L performs the analogous unwinding-and-loading role for the cytoplasmic exosome. This Exo-9 assembly is shared between compartments, but the catalytic subunits differ: DIS3 is predominantly nuclear, its paralog DIS3L is cytoplasmic, and EXOSC10 is predominantly nuclear, with abundant localization in nucleoli [32]. DIS3 is further largely excluded from the nucleolus, so nucleolar RNA surveillance is carried out predominantly by EXOSC10; in the cytoplasm the core instead associates with the SKI complex—in which the helicase SKIV2L replaces MTR4 and the adaptor SKI7 (a splice isoform of HBS1L) substitutes for MPP6—handing substrates to the exo-only nuclease DIS3L [39] (Figure 1c). The nuclear exosome engages a distinct substrate landscape: nascent noncoding transcripts, aberrant pre-mRNAs, and chromatin-associated regulatory RNAs, the discernment of which occurs via dedicated adaptor complexes discussed in the next section.

## 3. Adaptor Complexes: How the Exosome Finds Its Targets

The nuclear exosome lacks intrinsic means of discriminating substrates from non-substrates [16]. Substrate selectivity is instead conferred by MTR4-containing adaptor complexes (mTRAMP, NEXT, and PAXT), each tuned to different RNA classes.

The TRAMP (Trf4/5-Air1/2-Mtr4 Polyadenylation) complex was the first exosome adaptor identified, discovered simultaneously by three groups studying nuclear RNA quality control in *S. cerevisiae* [40,41,42]. The TRAMP complex appends a short poly(A) tail to aberrant transcripts via the non-canonical poly(A) polymerase Trf4, providing an unstructured 3′ extension that Mtr4 engages to feed substrates into the exosome [40]. The crystal structure revealed direct physical coupling of adenylation and unwinding [43]. TRAMP is the primary pathway for eliminating cryptic unstable transcripts (CUTs) [42].

**Figure 2:**
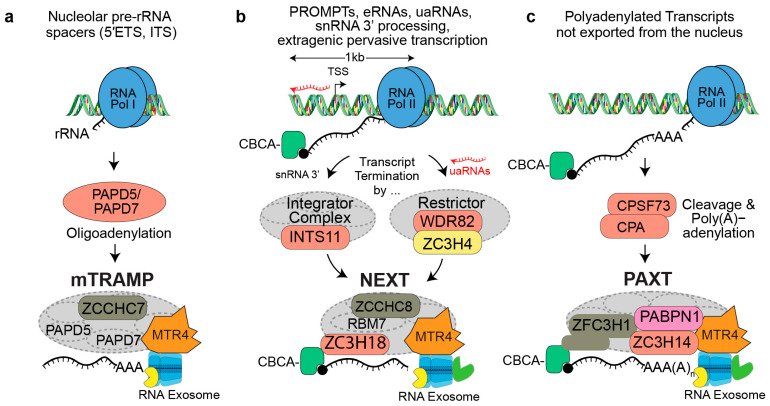
MTR4-containing adaptor complexes route distinct RNA classes to the nuclear exosome according to 3′-end identity. Each pathway couples a substrate-defining 3′ end (top) to a dedicated MTR4-based adaptor (bottom) that delivers the transcript to the shared, catalytically inert exosome barrel and its associated nucleases (DIS3 and EXOSC10/RRP6). (**a**) mTRAMP (nucleolar). RNA polymerase I-derived pre-rRNA processing intermediates and spacers (5′ETS, ITS) are oligoadenylated by the non-canonical poly(A) polymerases PAPD5/PAPD7, which append a short oligo(A) tag that serves as the degradation signal [33,40,41,42]. The mTRAMP adaptor (scaffold ZCCHC7 with PAPD5/PAPD7 and the helicase MTR4) delivers these uncapped substrates to the exosome; nucleolar localization, where DIS3 is largely excluded, favors EXOSC10. (**b**) NEXT (nucleoplasmic, pA−). Short, capped, unadenylated Pol II transcripts—PROMPTs, eRNAs, uaRNAs and snRNA 3′-processing products [1,45,46]—are terminated by the Integrator complex (INTS11 endonuclease [47]) or by the Restrictor complex (ZC3H4-WDR82), which enforces promoter directionality [48]. The released capped transcript, bound by the cap-binding complex-ARS2 (CBCA), is engaged by NEXT [56] via the bridging adaptor ZC3H18 [53] and degraded by the exosome. (**c**) PAXT (nucleoplasmic, pA+). Polyadenylated transcripts that fail to be exported—generated by cleavage and polyadenylation (CPSF73/CPA), which also licenses XRN2-mediated termination of the downstream product—are recognized through their long poly(A) tail. PAXT (scaffold ZFC3H1, which contacts CBCA directly; the poly(A)-binding protein PABPN1; the regulatory factor ZC3H14; helicase MTR4) targets these transcripts to the exosome and through ZFC3H1 retains polyadenylated substrates in the nucleus [57,58]. Structural analyses of the human NEXT and PAXT were recently performed [55,56] and ZFC3H1 (PAXT) and ZC3H18 (NEXT) compete for a common ARS2 epitope, biasing transcripts between the PAXT and NEXT routes [61]. Across all three pathways the exosome and MTR4 are invariant; specificity arises from the adaptor and the chemistry of the 3′ end. Adaptor-substrate assignments rest largely on factor depletion followed by RNA-seq, reporting steady-state accumulation rather than direct engagement, and the routes are not exclusive—loss of NEXT causes its substrates to be polyadenylated and captured by PAXT [60] so 3′-end identity is a strong bias rather than a strict determinant.

In *Schizosaccharomyces pombe*, a TRAMP-like complex containing Cid14 contributes to silencing of heterochromatic transcripts at centromeric repeats, acting in parallel with the RNAi pathway [3], establishing the first link between the exosome and epigenetic regulation. In mammalian nucleoli, a complex variant (mammalian TRAMP, mTRAMP) containing PAPD5 and ZCCHC7 monitors nucleolar RNA integrity [33] (Figure 2a). Mechanistically, this nucleolar complex writes its own destabilizing mark rather than reading a pre-existing one: PAPD5, together with its paralog PAPD7, appends a short, template-independent oligo(A) tract to pre-rRNA spacers and other aberrant nucleolar RNAs, and because DIS3 is largely excluded from the nucleolus these substrates are channeled preferentially to the EXOSC10 (RRP6) exonuclease [32,33]. This nucleolar activity is the exosome’s founding function in generating the mature 3′ end of 5.8S rRNAs [2] and cryo-EM of the nuclear exosome captured on a maturing pre-ribosome shows MTR4 docking directly onto the pre-ribosomal particle to deliver the 7S precursor into the barrel [44]. Because ribosomal RNA accounts for most transcriptome mass, pre-rRNA processing represents a large and continuous share of total exosome activity, a relevant point for later interpretation of exosomopathies (Section 6).

The Nuclear Exosome-Targeting complex (NEXT), comprising RBM7, ZCCHC8, and MTR4, is the principal adaptor for eliminating promoter upstream transcripts (PROMPTs) and enforcing transcription directionality [33,45,46] (Figure 2b). Two mechanistically distinct termination machineries generate the unadenylated 3′ ends that NEXT recognizes: the Integrator complex, whose INTS11 endonuclease cleaves nascent RNA at snRNA and promoter-proximal loci [47], and the catalytically inert Restrictor (ZC3H4-WDR82), which terminates unproductive transcription during early elongation by decelerating RNA polymerase II and acts as the principal enforcer of promoter directionality, preferentially terminating the upstream antisense (uaRNA) arm that lacks a U1-engaged 5′ splice site [48,49,50]. RBM7 binds broadly across Pol II-derived RNA co-transcriptionally [51], recognizing U-rich pyrimidine sequences [52], while the cap-binding complex serves as an upstream sorting platform [53]. Cryo-EM revealed that NEXT exists as a homodimer with two intertwined ZCCHC8 subunits arranging two MTR4 helicases; a C-terminal gatekeeping domain of ZCCHC8 encloses the RNA 3′ end within the MTR4 active site and must be displaced before exosome handover [54,55]. ZCCHC8 stimulates MTR4 helicase activity through a bipartite interaction distinct from yeast TRAMP cofactors [56]. The poly(A) tail exosome-targeting connection (PAXT), comprising MTR4, ZFC3H1, and PABPN1, is specialized for recognizing and degrading long polyadenylated noncoding transcripts [57,58] (Figure 2c). The poly(A) tails that mark these substrates are not added by PAXT itself but are generated in trans by the cleavage-and-polyadenylation (CPA) machinery—endonucleolytic cleavage by CPSF73 followed by canonical poly(A) polymerase—acting at canonical or premature/cryptic poly(A) sites, while the complementary downstream fragment is degraded by the XRN2 “torpedo” to terminate transcription; a single CPA event thus yields both the polyadenylated PAXT substrate and the XRN2-cleared termination product [59]. Additional components including ZC3H3, RBM26, and RBM27 are individually required for substrate turnover [60]. ZFC3H1 functions as a nuclear retention factor: in its absence, polyadenylated target transcripts escape to the cytoplasm via ALYREF (Aly/REF export factor, mRNA export adaptor of the TREX Transcription-Export complex) [58].

Recent work revealed that ZFC3H1 loads co-transcriptionally in a closed conformation that blocks export; on short, intron-poor transcripts, accessory factors trigger ZFC3H1 opening to license degradation, while on multi-exonic mRNAs, ALYREF displaces ZFC3H1 to permit export [61]. PAXT contains a TREX-2-like module (LENG8-PCID2-SEM1) that structurally mirrors the nuclear pore-associated export complex but instead promotes decay [62]. A “nuclear RNA degradation code” based on poly(A) tail length, splicing status, and 3′-end identity continues to be refined [63].

NEXT and PAXT activities are coordinated by the cap-binding complex-ARS2 (CBCA) sorting hub. The zinc finger protein ZC3H18 bridges the cap-binding complex to either NEXT or PAXT, channeling transcripts with heterogeneous 3′ ends to NEXT and those with poly(A) tails to PAXT [53,59,64,65]. Notably, ZC3H18 does not itself discriminate 3′-end status; the routing instead reflects the transcript’s end chemistry decoded by adaptor-specific subunits—most directly the recognition of the poly(A) tail by PABPN1 in PAXT—together with competition at a shared docking surface: the acidic ARS2-recruitment motifs of ZC3H18 and the PAXT scaffold ZFC3H1 engage a common ARS2 epitope in a mutually exclusive manner, such that ZC3H18 occupancy promotes NEXT while antagonizing PAXT [66]. When NEXT is depleted, its substrates become polyadenylated and are captured by PAXT, ensuring comprehensive surveillance [65]. ZC3H18 also couples exosome adaptors to transcription termination, interacting with the Integrator endonuclease and polyadenylation machinery [59]. Rapid depletion experiments refined NEXT substrate specificity, showing acute NEXT loss primarily affects snoRNA-hosting introns and short noncoding transcripts [67]. At the chromatin level, ZCCHC8, ZFC3H1, and MTR4 co-localize at transcription start sites and active enhancers, where they modulate cohesin binding and enhancer-promoter interactions [6]. The emerging picture is a highly organized surveillance network in which adaptors partition the noncoding transcriptome by 3′-end identity, compete for access to shared MTR4, and couple RNA fate to transcription termination and chromatin context [16,68].

## 4. The Exosome as an Epigenetic Effector: RNA Surveillance Across Chromatin States

The exosome actively participates in chromatin regulation, is physically present at transcription sites, and is functionally required for the maintenance of epigenetic states [69,70]. The first evidence that the exosome operates co-transcriptionally came from Drosophila, where exosome subunits associate with elongation factors and RNA Pol II at active loci on polytene chromosomes, with rapid recruitment to heat-shock-induced genes [69]. Genome-wide ChIP-seq revealed exosome occupancy at promoters and chromatin insulator elements bound by CP190, BEAF-32, and CTCF [70]. This enrichment at insulator boundaries suggested a functional relationship between RNA surveillance and the architectural proteins that delimit chromatin domains.

Critically, exosome-mediated RNA degradation is not merely a consequence of gene silencing but a prerequisite for it. In *S. pombe*, the TRAMP component Cid14 polyadenylates centromeric transcripts and channels them to the exosome; in cid14 mutants, reporter silencing is compromised even though H3K9me and Swi6/HP1 localization persist [3]. TRAMP-exosome and RNAi operate as parallel, partially redundant pathways for heterochromatin nucleation [71]. RNA clearance is therefore an instructive step in establishing and maintaining repressive chromatin, not a passive cleanup afterward.

**Figure 3:**
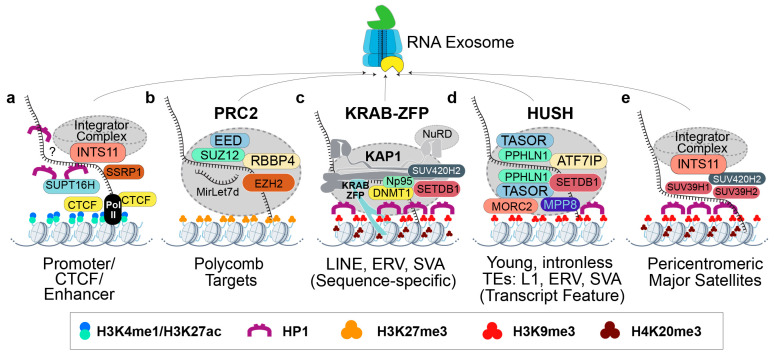
Chromatin-state-specific routes feed nascent transcripts to the nuclear RNA exosome. The nuclear RNA exosome acts as a single convergent effector that is recruited to chromatin through divergent, state-specific routes. (**a**) Active promoters, CTCF/insulator sites, and enhancers (H3K4me3/H3K4me1/H3K27ac; H3K9me3-independent). HP1 is recruited independently of H3K9me3 [10], including via the ChAHP (ADNP-CHD4-HP1) complex as an H3K9me3-independent route [78]. Here the adaptor ZC3H18 couples the elongating Pol II/FACT machinery (SUPT16H, SSRP1) to RNA decay [64]. Short, poly(A)-deficient PROMPTs and enhancer RNAs are terminated by the Integrator or Restrictor complex and routed through NEXT to the exosome, tuning regulatory-RNA levels without depositing a repressive mark. (**b**) Polycomb (PRC2) targets (H3K27me3; bidirectionally transcribed). In the MiCEE assembly, the microRNA Mirlet7d recognizes nascent ncRNA and recruits the exosome cofactor C1D/RRP47 [81], which simultaneously engages the exosome via EXOSC10/RRP6 and the PRC2 complex: catalytic methyltransferase EZH2, activator EED, scaffold SUZ12, and chaperone RBBP4. (**c**) In KRAB-ZFP-based repression, a KRAB zinc finger protein recognizes the DNA sequence of the transposable element (such as an endogenous retrovirus (ERV) or its truncated long terminal repeat (LTR), or LINE or SVA element). The KRAB-ZFP then recruits KAP1/TRIM28, which engages SETDB1, the NuRD complex, the DNA-methylation machinery (UHRF1/Np95, DNMT1), and SUV420H2 for H4K20me3 deposition (reviewed in [82]). Full-length, poly(A)+ ERV/LTR transcripts are routed via PAXT (ZFC3H1) to the exosome while smaller poly(A)− ERV transcripts may be routed by NEXT. Here HP1 recruits the exosome to LTR transcripts through direct protein-protein contact, independently of HUSH [10]. (**d**) HUSH selects targets by transcript length and intronlessness rather than sequence [83], distinguishing it from the KRAB-ZFP route in (**c**); it enables silencing of young full-length LINE-1s, SVAs, and a subset of LTR/ERVs which are defined by RNA features, not sequence. HUSH (TASOR, Periphilin/PPHLN1, MPP8) recruits SETDB1 (with ATF7IP) and the compaction ATPase MORC2; Periphilin reads the nascent transcript to initiate silencing, and MPP8 subsequently reads the deposited H3K9me3 to maintain it. Short, poly(A)-deficient transposable element transcripts are degraded via NEXT (ZCCHC8) [8]. (**e**) Pericentromeric major satellites (H3K9me3, H4K20me3). SUV39H1/SUV39H2 deposit H3K9me3 read by HP1 (SUMO-HP1α seeds de novo targeting via the forward satellite transcript), and SUV420H2 deposits H4K20me3. Low-level, cap-less and poly(A)-deficient satellite transcripts are attenuated by the Integrator complex [84], which generates the unadenylated 3′ ends funneled through NEXT to the exosome; because the same transcript is instructive for nucleating HP1, surveillance calibrates rather than abolishes it.

The RNA exosome is enriched over at least five distinguishable chromatin states, each with a characteristic recognition module but a shared degradative output (Figure 3): (i) active promoters, CTCF/insulator sites, and enhancers; (ii) H3K27me3 Polycomb chromatin; and three flavors of H3K9me3 heterochromatin—(iii) KRAB-KAP1-directed ERVs and LTRs, (iv) HUSH-directed young LINE-1 elements, and (v) pericentric satellites.

### 4.1. Heterochromatin Protein 1 Is a Principal Mammalian Exosome Adaptor

How the exosome is directed to chromatin in mammalian cells depends largely on Heterochromatin Protein 1 (HP1) [10,72], as it appears to be involved in four out of the five chromatin states where the exosome is active. Mammalian genomes encode three HP1 paralogs, HP1α (CBX5), HP1β (CBX1), and HP1γ (CBX3), each containing a chromodomain that reads H3K9me2/3 and a chromoshadow domain for dimerization. HP1α requires both its chromodomain and an RNA-binding hinge region for heterochromatin targeting; mutation of three conserved lysines abolishes both RNA binding and pericentromeric localization, and RNase treatment disperses HP1 from foci [73,74].

HP1γ additionally binds intronic repeat sequences in pre-mRNAs and modulates alternative splicing at active genes [75,76], illustrating that HP1-RNA contacts are not restricted to heterochromatin. The mechanistic logic of HP1-RNA coupling was first elucidated in *S. pombe*. Swi6, the HP1 homolog in this species, captures centromeric noncoding RNAs, and RNA competes with H3K9me for chromodomain binding, creating a “heterochromatin checkpoint”: Swi6 molecules capture aberrant transcripts, RNA binding ejects Swi6 from chromatin, the transcript is degraded, and only then does Swi6 re-engage H3K9me [72,77].

Direct evidence that mammalian HP1 recruits the exosome came from triple HP1 knockout (HP1TKO) in murine embryonic liver (BMEL) cells [10]. Co-immunoprecipitation confirmed that HP1β and HP1γ associate with EXOSC9, EXOSC10, MTR4, and ZC3H18; critically, these interactions are RNase-resistant, indicating protein-mediated contacts rather than RNA-bridged associations, distinguishing this mammalian mechanism from the RNA-dependent Swi6 pathway. Loss of all three HP1 isoforms causes EXOSC9 and EXOSC3 to shift from the nucleus to the cytosol, demonstrating that HP1 is required not only for chromatin targeting but for nuclear retention of core exosome subunits [10]. HP1TKOs further show a dramatic reduction in MTR4, ZC3H18, and EXOSC10 ChIPseq signal, with a reciprocal increase in RNA Pol II occupancy. Importantly, HP1- and exosome-mediated surveillance operates in two mechanistically distinct modes: an H3K9me3-independent mode at promoters, CTCF sites, and enhancers, and an H3K9me3-dependent mode at repeats [10]. The following sections trace the exosome through each chromatin state along this divide.

### 4.2. The Exosome at Promoters, CTCF Sites, and Enhancers

At transcription start sites, HP1 and exosome enrichment is independent of H3K9me3 [10], in striking contrast to the H3K9me3-coupled HP1 enrichment seen at repeats. Notably, 90% of MTR4 peaks overlap with CTCF binding sites, and HP1 concentrates both MTR4 and CTCF at a subset of promoters, directly connecting exosome recruitment to insulator biology. How HP1 itself is targeted to these H3K9me3-independent promoters and CTCF-associated sites remains unresolved. It may occur via direct binding of small RNAs to the HP1 hinge domain, or via HP1-mediated recruitment of the exosome through an accessory complex. A prime candidate is the ChAHP (ADNP-CHD4-HP1) complex, a known H3K9me3-independent mechanism of HP1 recruitment [78] (Figure 3a). A possible link to RNA surveillance is that the exosome adaptor ZC3H18 itself co-purifies with transcription elongation subunits SSRP1 and SUPT16H and with HP1/CBX proteins [64]. However, a direct connection between ChAHP and the exosome remains unestablished, and the primary evidence indicates that ChAHP typically competes with rather than coexists with CTCF [79]. At enhancers, the same logic applies: HP1 loss stabilizes enhancer RNAs, activating enhancers near collagen genes (*Col6a1*, *Col6a2*), a sensitivity independently confirmed in EXOSC3-knockout ESCs and ZFC3H1-depleted HeLa cells [10]. This mirrors the established role of the mammalian exosome in degrading enhancer and super-enhancer RNAs to control their activity [80]. Together, these observations indicate that at active regulatory elements the exosome functions less to silence loci outright than to set the steady-state level of regulatory noncoding RNA.

### 4.3. The Exosome at H3K27me3 (Polycomb) Chromatin

The exosome also interacts with Polycomb-based repression. Once termed the MiCEE complex, named for containing microRNA Mirlet7d, the exosome cofactor C1D/RRP47, EXOSC10, and EZH2 (the catalytic methyltransferase of PRC2), it cooperates to initiate RNA-based epigenetic repression. Here, Mirlet7d recognizes noncoding RNAs at bidirectionally active loci and recruits C1D, which simultaneously engages the exosome and PRC2, coupling RNA decay to H3K27me3 deposition and perinucleolar tethering [81]. This coupling is bidirectional: loss of the PAXT component ZFC3H1 causes global accumulation of nuclear poly(A)+ RNA that is proposed to titrate PRC2 away from chromatin, reducing H3K27me3 and blocking differentiation [5]. Recent evidence extends this titration model to the NEXT arm of the pathway and identifies the RNA features on both sides of the interaction. Nascent long noncoding RNAs bearing G-quadruplex (G4) motifs are bound directly by EZH2, which blocks recruitment of PRC2 to chromatin, while the same transcripts carry U-rich motifs read by RBM7 that route them to the exosome for degradation [85]. Loss of ZCCHC8 accordingly stabilizes these G4/U-rich lncRNAs and lowers both EZH2 occupancy and H3K27me3 genome-wide, whereas ZCCHC8 overexpression raises H3K27me3. This effect is specific to H3K27me3, since H3K27ac, H3K4me3 and H2AK119ub1 are unchanged, and one that occurs without any alteration in PRC2 subunit levels or complex integrity [85]. In clear-cell renal cell carcinoma and lung adenocarcinoma, high ZCCHC8 expression drives excessive degradation of these transcripts, liberating PRC2 to deposit H3K27me3 at neighboring loci and silence adjacent tumor suppressors including *SEMA5A* and *ARID1A*, and predicting greater sensitivity to the EZH2 inhibitor tazemetostat [85]. Nuclear RNA decay therefore sets the free concentration of PRC2 available to chromatin, with the exosome acting as a rheostat rather than a switch.

These titration models require one methodological caveat. Much of the evidence that RNA controls PRC2 chromatin association derives from degrading RNA with RNase A during chromatin immunoprecipitation, and this assay has now been shown to mislead: RNA degradation precipitates chromatin out of solution and produces a gain of non-targeted DNA that artifactually depresses immunoprecipitation signal at facultative heterochromatin after normalization [86], an effect reversed by maintaining chromatin solubility with poly-L-glutamic acid [87]. Support for a model in which RNA physically bridges PRC2 to chromatin is correspondingly weakened. The exosome-Polycomb link nonetheless stands, because it rests on orthogonal genetic evidence: loss of ZFC3H1 or ZCCHC8 alters H3K27me3 and PRC2 occupancy measured without RNase treatment [5,85]. The requirement is conserved from *Caenorhabditis elegans* where RNA-processing machinery including the LSM2-8 complex (a nuclear, hetero-heptameric ring of seven Sm-like proteins, LSM2-LSM8, canonically required for binding the 3′ end of U6 snRNA in splicing) silences H3K27me3-marked loci through targeted RNA decay. This occurs via the 5′→3′ exonuclease XRN-2 rather than the exosome, a parallel nuclear surveillance route [4].

An epitranscriptomic layer further connects these pathways: m^6^A modification of chromosome-associated regulatory RNAs (carRNAs) marks these transcripts for decay through the NEXT-exosome pathway once they are read by the principal nuclear m^6^A reader YTHDC1, and loss of the m^6^A writer METTL3 stabilizes them and increases chromatin accessibility [88]. The same epitranscriptomic route operates on the archetypal facultative-heterochromatin system. Rapid degron depletion has shown that the principal function of m^6^A on Xist, the noncoding RNA that initiates X-chromosome inactivation and recruits Polycomb to the inactive X, is to license its turnover by the NEXT complex: acute loss of METTL3 stabilizes Xist and accelerates Xist-mediated silencing, and acute loss of ZCCHC8 does so more strongly still, while depletion of the PAXT subunit ZFC3H1 does not [89]. Metabolic labeling attributes the elevated Xist to increased transcript stability rather than increased synthesis, and the turnover proceeds independently of YTHDC1, indicating that the reader requirement differs between carRNAs and Xist [89]. The imprinted Polycomb-recruiting transcript *Kcnq1ot1* behaves likewise [89]. Exosome surveillance therefore does not merely remove a silencing lncRNA; it sets the kinetics with which that lncRNA establishes a Polycomb-marked domain.

Whether these effects reflect a circuit dedicated to PRC2 remains unsettled. Degron-mediated depletion of the core subunit EXOSC2 in embryonic stem cells triggers acute nuclear RNA aggregation that sequesters nuclear proteins and suppresses transcription globally, yet at early timepoints chromatin accessibility rises while H3K4me3, H3K27ac and H3K27me3 remain unchanged, with heterochromatin reorganization emerging only after a cell cycle [22]. Distinguishing a dedicated exosome-Polycomb circuit from PRC2 being one casualty of a broader collapse in nuclear RNA-protein stoichiometry is, in our view, the central unresolved question at this chromatin state.

### 4.4. The Exosome at KRAB-KAP1 Heterochromatin (ERVs and LTRs)

Within H3K9me3-positive heterochromatin, the exosome is recruited through at least three partially independent arms that converge on RNA clearance (Figure 3c–e). The first centers on the canonical retroelement-silencing pathway. The canonical heterochromatin pathway at transposable elements relies on KRAB zinc finger proteins to recruit KAP1 (TRIM28), which engages SETDB1 to deposit H3K9me3 and silence transposable elements, prominently endogenous retroviruses [90]. In human neural progenitor cells, individual KRAB-ZFPs such as ZNF91 establish mini-heterochromatin domains over SVA insertions that constrain their cis-regulatory impact on neighboring genes; loss of these domains aggravates X-linked dystonia-parkinsonism pathology [91]. The exosome is recruited to this class of elements through HP1 rather than through KAP1 directly. ERVs and their respective long terminal repeats (LTRs) are de-repressed in HP1-deficient cells, where chimeric transcription becomes abundant [9,10]. In HP1TKO cells, transcription of elements such as RLTR17 and RMER13A extends beyond annotated repeat boundaries and the transcripts appear spliced in the cytoplasm [10], displaying canonical mRNA maturation after escape from nuclear exosome surveillance. These LTR transcripts overlap with those stabilized upon PAXT (ZFC3H1) or NEXT (ZCCHC8) depletion in ESCs [8] confirming impaired exosome-mediated degradation as the mechanism. HP1 thus channels these transcripts to the exosome through direct protein-protein contact rather than by reinforcing transcriptional silence; indeed, HP1 does not directly repress these elements but instead promotes degradation of RNAs transcribed from LTRs and enhancers via exosome recruitment [10].

Because each KRAB-ZFP must evolve to recognize a specific DNA sequence [92,93], newly integrated retroelements can transiently evade this pathway until a cognate ZFP arises. This gap is filled by a sequence-independent epigenetic defense, the RNA-directed Human Silencing Hub (HUSH). The KRAB-KAP1-HP1-exosome arm is mechanistically distinct from the HUSH pathway: the LTR loci that accumulate transcripts upon HP1 triple knockout do not overlap with the MPP8/HUSH-regulated loci identified in mESCs [10], dual-pathway architecture to direct retroelement RNA to the exosome, one that is sequence-dependent (KRAB-KAP1-HP1) and one that is not (HUSH).

### 4.5. The Exosome at HUSH-Directed Heterochromatin

HUSH fills the gap left by sequence-specific KRAB-ZFPs as an RNA-directed, sequence-independent silencing system. HUSH selects long, intronless, highly transcribed RNAs such as recently mobilized elements like full-length LINE-1s and retrocopies, while introns and efficient splicing protect host transcripts from repression [83,94]. Because most cellular mRNAs are spliced and rapidly exported, they escape surveillance, whereas intronless, chromatin-retained transcripts dwell at their site of synthesis long enough to be captured. Targeting is thus kinetic and feature-based, which is how a broadly RNA-binding complex achieves selectivity without a cognate DNA sequence.

Critically, silencing is initiated by the RNA-binding subunit Periphilin (PPHLN1), which engages the nascent transcript via its N-terminal domain, dimerizing and localizing the TASOR scaffold together with MPP8 to the transcribed locus. Here the chromodomain of MPP8 [95] recruits the H3K9 tri-methyltransferase SETDB1 and its cofactor ATF7IP to initiate nucleation [96,97]. This RNA recognition is the limiting step shown by tethering experiments: artificially anchoring Periphilin to a transcript bypasses the need for its RNA-binding domain yet still triggers H3K9me3 deposition and silencing [98]. After initial nucleation, MPP8’s chromodomain binds the newly deposited H3K9me3, stabilizing HUSH and recruiting further SETDB1 to propagate the mark, in a similar feed-forward manner as canonical SETDB1-HP1 repression.

In mouse ESCs, HUSH recruits NEXT to MPP8-bound TE loci, where NEXT targets shorter non-polyadenylated TE transcripts for exosomal degradation while HUSH transcriptionally suppresses full-length polyadenylated TEs. ZCCHC8 knockout in ESCs upregulated 10,202 TE RNAs spanning all three retrotransposon classes, whereas ZFC3H1 knockout produced a weaker, predominantly LTR-directed effect (1705 upregulated TEs). In vivo, ZCCHC8-knockout mice exhibit LINE-1 de-repression with H3K9me3 loss, failed chromatin condensation during spermatogenesis, and male subfertility [99].

Importantly, HUSH and the KRAB-KAP1 pathway are not entirely mutually exclusive: KAP1/TRIM28 co-occupies a subset of HUSH target loci, where the two systems cooperate to repress young retrotransposons. Their co-occupancy reflects convergence on the shared SETDB1/H3K9me3 [100]. This convergence is understandable as the genome adapts from a transcription-based repression (HUSH) to a more permanent, sequence-specific (KRAB-ZFP-based) repression.

### 4.6. The Exosome at Pericentric Satellites

The third H3K9me3 arm operates at pericentromeric major satellite repeats (MaSat/MSR), where constitutive heterochromatin is assembled by a hierarchical, self-reinforcing circuit of writers and readers (Figure 3e).

Pericentric transcription is therefore instructive rather than incidental. SUMO-1-modified HP1α associates specifically with forward-strand satellite transcripts and requires this interaction for de novo targeting to pericentric foci [101], and major satellite RNA stabilizes the retention of SUV39H on chromatin through RNA-nucleosome association and RNA:DNA-hybrid formation [102]. The methyltransferases SUV39H1 and SUV39H2 deposit H3K9me3, which is bound by HP1 through its chromodomain; HP1 then both recruits additional SUV39H to propagate the mark in the classical read-write loop, and recruits SUV420H2 (KMT5C) to add H4K20me3, the second constitutive-heterochromatin mark [103,104]. Consistent with an instructive role, satellite transcription is required for chromocenter formation in early mouse development [105], and isolated MSR units relocate to an inert, gene- and repeat-free locus nucleate H3K9me3 and recruit HP1 only when they are transcriptionally competent [84]. In other words, the transcript seeds the HP1-SUV39H circuit that the read-write loop subsequently maintains.

These nucleating transcripts are bidirectional, cap-less, and poly(A)-deficient, remain chromatin-retained, and engage initiating but not elongating (Ser2-phosphorylated) RNAPII, and are the posterchild of promoter-proximally terminated, non-productive transcription [84]. They are attenuated by the Integrator complex, whose endonuclease INTS11 (with INTS12) is enriched at competent MSR units and generates the unadenylated 3′ ends funneled through NEXT to the exosome; INTS11 depletion raises intact-MSR transcript levels several-fold and modestly lowers HP1α without changing H3K9me3 [84]. This places satellite surveillance alongside the HP1-recruited exosome at LTRs: both act on bidirectional, poly(A)-deficient repeat RNA and selectively tune the HP1 layer rather than H3K9me3. Crucially, because satellite-RNA overaccumulation disrupts heterochromatin [106], this surveillance calibrates rather than abolishes an instructive transcript, holding it within a permissive window. Two caveats temper this current model: Integrator, like RNAPII, is general machinery distributed broadly across ERVs, genes, and regulatory elements rather than a dedicated heterochromatin factor; whether the HP1α loss upon INTS11 depletion reflects disrupted protein-protein contacts or simply elevated RNA output remains unresolved [84].

## 5. RNA Surveillance and 3D Genome Organization

Exosome-mediated RNA turnover is an active determinant of higher-order genome architecture [6,7]. By clearing noncoding RNAs at promoters, enhancers, and insulators, the exosome directly influences three-dimensional chromatin organization.

Mammalian Pol II promoters are inherently bidirectional, and PROMPTs—short, unstable upstream antisense transcripts—are cleared by the nuclear exosome through two adaptor routes that together impose apparent unidirectionality on a fundamentally symmetric process: NEXT captures short, non-polyadenylated species co-transcriptionally [33], while premature polyadenylation at upstream poly(A) sites marks others for PAXT-dependent decay and is the step that enforces promoter directionality [1,46]. U1 snRNP reinforces this by suppressing premature polyadenylation of sense mRNAs and regulating chromatin retention of noncoding RNAs [45,107]. Beyond promoters, the exosome co-localizes with the insulator proteins CTCF, CP190, and BEAF-32 [69,70], and at super-enhancers, the exosome is the principal agent of eRNA turnover; conditional knockout of EXOSC3 and EXOSC10 uncovered thousands of exosome-sensitive eRNAs, including lncRNA-CSR at the immunoglobulin heavy chain super-enhancer, linking exosome-controlled eRNA abundance to enhancer output [80,108].

The exosome’s architectural reach extends to loops and domains through its adaptors. ZCCHC8 (NEXT), ZFC3H1 (PAXT), and MTR4 occupy sites of enhancer-promoter interaction, and depleting any of them increases noncoding RNA accumulation and cohesin binding there. MTR4 loss specifically strengthens loop-anchor contacts while reducing intraloop contacts, indicating that MTR4-dependent RNA clearance promotes processive cohesin-mediated loop extrusion [6]. At the Igh locus, the exosome associates with AID and is required for AID access to switch-region DNA [109]. Exosc3 knockout impairs class-switch recombination through accumulation of divergently transcribed xTSS-RNAs [108]. DIS3 inactivation causes noncoding RNA accumulation at CTCF-binding elements, decreased CTCF occupancy, disorganized cohesin, disrupted TAD integrity, genome-wide R-loop accumulation, and chromosomal translocations [7]. Consistent with an R-loop resolving role, EXOSC10 depletion causes R-loop accumulation at DNA double-strand breaks and impairs homologous recombination [110], and the MTR4 DNA:RNA-hybrid helicase restricts AID-mediated mutational asymmetry at Igh [111].

### The Protocadherin Cluster: Where Heterochromatin, Antisense RNA, and the Exosome Converge

The preceding sections treated the exosome’s chromatin functions separately: adaptor-dependent substrate choice, HP1- and HUSH-directed heterochromatin, CTCF-based insulation, and clearance of antisense and noncoding RNA. In vivo these are coupled, and testing their interplay requires a locus where all are simultaneously active, individually measurable, and tied to a phenotype. The clustered protocadherin locus meets all three. Its alternate promoters begin in a heterochromatic ground state, so silencing and its release are directly scorable; isoform choice is stochastic and allele-specific, giving a single-cell readout; and that choice is initiated by an antisense lncRNA subject to RNA surveillance and executed through CTCF/cohesin contacts between the chosen promoter and a distal enhancer, mechanistically coupling RNA turnover, heterochromatin, and 3D architecture. Like olfactory and vomeronasal receptor genes, they achieve stochastic monogenic expression through H3K9me3-based silencing [112]. Here antisense transcription is not noise but the initiating event of a gene-choice mechanism that assigns each neuron a unique cell-surface identity code, placing the locus at the intersection of antisense RNA surveillance and heterochromatin silencing.

Protocadherin isoform choice operates per cell and per allele, with each Pcdh-α allele expressing only one or two isoforms [113,114]. Prior to isoform selection, the repressed ground state is defined by CpG methylation at CTCF sites and H3K9me3 across the alternate promoter array [114,115,116]. Here H3K9me3 deposition occurs via SETDB1 [117], which is thought to be recruited by HUSH; conditional knockout of MPP8 or MORC2A de-represses the cluster in both mouse brain and human cerebral organoids [118]. Notably, brains deficient in HP1γ also show marked upregulation of protocadherin isoforms [9], suggesting that here HUSH and HP1 systems converge. Given that (antisense) lncRNAs are important in triggering H4K20me3-based transcriptional silencing [119], and that H4K20me3 is lost upon disruption of HP1γ [9], we hypothesize that HP1γ specifically cooperates with SUV420H2, HUSH components, and the RNA exosome in coordinating protocadherin selection. A prerequisite for this to occur is prior recruitment of G9a and GLP/EHMT1 by Wiz to deposit H3K9me1/2 at CTCF sites [120]. Clinically, haploinsufficiency of GLP/EHMT1 results in Kleefstra syndrome, where excess conversion to H3K9me3 by SETDB1 across the locus accompanies cognitive dysfunction [121,122].

Isoform selection occurs when an antisense lncRNA breaks through this heterochromatin. Stochastic firing of an antisense promoter within an alternate exon produces a polyadenylated nuclear lncRNA, the substrate class surveyed by PAXT that traverses the sense promoter and recruits TET3 to demethylate the flanking CTCF sites, licensing CTCF binding and cohesin-mediated enhancer-promoter looping [113,116,123]. Here the cohesin unloader WAPL functions as a rheostat: high WAPL biases expression toward proximal isoforms, while low WAPL enables diverse distal isoform expression, generating deterministic versus stochastic Pcdh repertoires across cell types [124]. Cohesin extrusion trajectories, CTCF boundary strength, and enhancer identity combinatorially determine promoter contact probabilities [114].

Combinatorial Pcdh expression underlies dendritic self-avoidance [125] and olfactory neural circuit assembly [126], and, through interaction with Neuroligin-1, negatively regulates excitatory synaptogenesis [127]. The clinical urgency is underscored by pontocerebellar hypoplasia (EXOSC3, EXOSC8, EXOSC9 mutations) devastating precisely the tissues where Pcdh self-avoidance is critical and HUSH normally restricts isoform diversity [118,128,129,130]. Whether Pcdh diversity is in fact perturbed in exosomopathy models has not been examined.

## 6. Exosomopathies

The term “exosomopathy” [18] encompasses a growing family of Mendelian disorders caused by mutations in exosome subunits and adaptor components, with many producing pathologies with tissue sensitivity. Perturbation of core components produces extreme phenotypes, such as the DIS3L knockout, which causes embryonic lethality around E6.5 [131]. Inhibition of other components results in the nervous system being most sensitive; however, pathology also extends to muscle, lung, kidney, gut, and hematopoietic tissues. A comprehensive description of exosome-, adaptor-, and epigenetic-related genes along with known human disease associations is provided in Table 1.

Pontocerebellar hypoplasia type 1 (PCH1) is a group of autosomal recessive neurodegenerative disorders characterized by cerebellar hypoplasia, progressive microcephaly, and anterior horn cell degeneration. PCH1b is caused by EXOSC3 mutations (cap subunit Rrp40), with patients showing severe cerebellar hypoplasia and spinal motor neuron loss [128]. PCH1c results from EXOSC8 mutations (ring subunit Rrp43) and is distinguished by prominent CNS hypomyelination [129]. PCH1d is caused by EXOSC9 variants (ring subunit Rrp45), with patient fibroblasts showing reduced EXOSC9 protein and destabilized exosome assembly [130]. Additional subtypes include PCH1f [132], EXOSC5 variants [133], and a biallelic EXOSC4 variant (p.Leu187Pro) extending the spectrum to the core ring [134]. Di Donato and colleagues reported EXOSC2 mutations causing a distinct multi-system syndrome with retinitis pigmentosa, hearing loss, and premature aging [135], illustrating how different barrel positions produce strikingly different presentations. Among the PCH subtypes (PCH1b-1d, PCH1f), the cerebellum is universally affected and spinal motor neurons are involved in most, presumably reflecting their high transcriptional output [128,129,130].

Recently, exosome dysfunction was profiled at single-cell resolution by Higginson et al. (2026) [136], who generated Drosophila PCH1b mutant models carrying pathogenic EXOSC3/Rrp40 mutations and profiled 116,619 nuclei across 25 cell types. The severe G11A allele dysregulated 2537 transcripts, and the milder G146C allele dysregulated 1743, with most increased in abundance. Notably, the domesticated retrotransposon Gag protein Arc1, which regulates synaptic plasticity via retrovirus-like capsids, expanded from 6% to 83% of cells in severe mutants. Arc1 overexpression alone produced age-dependent mushroom body morphology defects. Progressive mushroom body degeneration, locomotor decline, and memory impairment were rescued by pan-neuronal expression of human EXOSC3, confirming functional conservation across hundreds of millions of years of evolution. The key finding is that individual cell types vary in their reliance on RNA surveillance, with Kenyon cells (mushroom body neurons) emerging as particularly vulnerable.

It must be noted that core EXOSC mutations do not remove a pathway but lower the capacity shared by every exosome activity at once; because pre-rRNA processing imposes the largest continuous draw on that capacity, the deficit surfaces first as impaired ribosome biogenesis and translation [22,134], and therefore first in the cells of highest biosynthetic output: the cerebellar and motor neuron bias of PCH, and the Kenyon cell vulnerability in Drosophila [136]. Mutations in the nuclear cofactors instead act on specificity: NEXT and PAXT are dispensable for nucleolar processing, so their loss spares ribosome biogenesis and withdraws surveillance only from the defined nucleoplasmic chromatin-associated transcripts of Section 4. Diseases associated with exosome cofactors consequently are not concentrated in one vulnerable cell class but distributed across tissues according to which adaptor is limiting.

Mutations in NEXT and PAXT components severely impact cellular function across cell types. Heterozygous ZCCHC8 loss-of-function causes familial pulmonary fibrosis through impaired 3′-end processing of telomerase RNA (TERC), leading to telomere shortening and alveolar stem cell exhaustion [137]. Dysregulated RBM7 expression drives fibrotic remodeling through enhanced degradation of NEAT1, apoptosis, and recruitment of pro-fibrotic atypical monocytes [138]. An RBM7 missense mutation (p.Pro79Arg) causes SMA-like spinal motor neuropathy [139]. Oculopharyngeal muscular dystrophy (OPMD), caused by GCG repeat expansions in PABPN1, represents a PAXT-linked exosomopathy: PABPN1 aggregation in muscle fibers is predicted to impair PAXT-mediated poly(A)+ ncRNA surveillance [58,140,141,142]. Notably, gain-of-function MORC2 mutations (p.R252W) hyperactivate HUSH-mediated silencing at approximately 7.5-fold lower expression, causing Charcot-Marie-Tooth disease type 2Z, indicating that excessive heterochromatin compaction is as pathogenic as its loss [143]. The RNA exosome is also implicated in C9orf72-ALS/FTD, where EXOSC10 degrades expanded repeat RNA; arginine-rich dipeptide repeat proteins inhibit EXOSC10, establishing a feed-forward cycle of repeat RNA accumulation and exosome impairment [144]. Beyond the core exosome, DIS3L2 mutations cause Perlman syndrome of developmental overgrowth and Wilms tumor predisposition [145], and SKIV2L mutations cause trichohepatoenteric syndrome [146].

**Table 1:** Exosome-related genes, complexes, and human disease associations.

Gene	Aliases	Complex/ Context	Function	Mendelian Disease (OMIM)	Other Disease Associations
**Core Exosome—Cap Subunits**					
*EXOSC1*	CSL4	Exo-9 cap	Cap subunit	PCH type 1F; p.Arg183Trp (619304) [132]	Dilated cardiomyopathy [132]; KO mice: embryonic lethality (gastrulation failure by E7.5; [147])
*EXOSC2*	RRP4	Exo-9 cap	Cap subunit; depletion triggers pre-senescence	SHRF syndrome (617763) [135]	Loss of pluripotency in ESCs [22]; KO mice: peri-implantation lethality [147]
*EXOSC3*	RRP40	Exo-9 cap	First exosome gene linked to disease	PCH type 1B (614678) [128]	Impaired CSR/SHM; senescence upon depletion [136]; zebrafish/Drosophila KD models; Drosophila G11A: Arc1 in 83% of cells, mushroom body degeneration
**Core Exosome—Ring Subunits**					
*EXOSC4*	RRP41	Exo-9 ring	PH-like ring subunit	Neurodevelopmental disorder (AR) [134]	Amplified in multiple cancers [148]
*EXOSC5*	RRP46	Exo-9 ring	Ring subunit	CABAC (619576) [133]	
*EXOSC6*	MTR3	Exo-9 ring	PH-like hexamer	None reported	
*EXOSC7*	RRP42	Exo-9 ring	PH-like hexamer	None reported	
*EXOSC8*	RRP43	Exo-9 ring	Knockdown disrupts myelin mRNA	PCH type 1C (616081) [129]	Zebrafish KD: motor neuron defects, hypomyelination [129]
*EXOSC9*	RRP45	Exo-9 ring	Mutations destabilize exosome	PCH type 1D (618065) [130]	Zebrafish KD: cerebellar/hindbrain loss, motor neuron defects [130]
**Catalytic Subunits**					
*DIS3*	RRP44	Nuclear exosome	3′→5′ exo+endonuclease	Familial MM susceptibility (607533) [21]	Somatic MM 10–18.5%; tumor suppressor [19,20]; TAD disruption via DNA:RNA hybrids, AID-driven translocations; hematopoietic KO: pancytopenia
*DIS3L*	DIS3L1	Cytoplasmic exosome	Cytoplasmic 3′→5′ exoribonuclease	None reported	Embryonic lethality in KO mice [131]
*DIS3L2*	—	Exosome-independent	Degrades uridylated RNAs	Perlman syndrome (267000) [145]	KO mice partially phenocopy Perlman syndrome: GU defects, Igf2 upregulation [149]
*EXOSC10*	RRP6	Nuclear exosome	Distributive 3′→5′ exoribonuclease	None reported	PM/Scl autoantigen; HCC; ALS/FTD [144,150,151]; KO: embryonic lethality (morula arrest); germ cell cKO: infertility both sexes [152]
**Core-Proximal Cofactors**					
*C1D*	RRP47	Nuclear exosome	Stabilizes EXOSC10; MiCEE bridge	None reported	
*MPP6*	—	Nuclear exosome	Tethers MTR4 to exosome	None reported	
*SKIV2L*	SKI2	Cytoplasmic SKI	Helicase; threads mRNA into exosome	THES2 (614602) [146]	
*TTC37*	SKI3	Cytoplasmic SKI	SKI complex component	THES1 (222470) [146]	
**NEXT Complex**					
*MTR4*	MTREX, SKIV2L2	NEXT/PAXT/TRAMP	Universal exosome helicase	None reported	Essential for all adaptor pathways; KO: embryonic lethality (E6.5); germ cell cKO: male infertility, meiotic initiation failure
*ZCCHC8*	—	NEXT scaffold	Bridges NEXT to HUSH; TERC maturation	PFBMFT5 (618674) [137]	Postnatal neuropathology; subfertility; ROS1 fusion; L1 de-repression [55,99,137]; KO mice: fatal ciliopathy-like neurodevelopmental pathology; het: TR insufficiency
*RBM7*	—	NEXT RNA-binding	Binds Pol II RNA; promotes NEAT1 degradation	SMA-like neuropathy; p.Pro79Arg [139]	Fibrosis: upregulated; knockdown suppresses [138]
**PAXT Complex**					
*ZFC3H1*	—	PAXT scaffold	Nuclear retention factor	None reported	Pan-cancer biomarker; PRC2 destabilization [5]
*PABPN1*	—	PAXT	Poly(A)-binding protein	OPMD1 (164300) [140]	Intranuclear aggregates in muscle [140]; multiple mouse models recapitulate OPMD: knockin, transgenic, inducible (progressive myopathy)
*ZC3H3*	—	PAXT accessory	Triggers ZFC3H1 opening	None reported	
*RBM26*	—	PAXT accessory	Required for substrate turnover	None reported	
*RBM27*	—	PAXT accessory	Required for substrate turnover	None reported	SFARI autism candidate (5 de novo variants)
*ZC3H14*	—	PAXT regulatory	Antagonizes PABPN1; CDK13 substrate	MRT56: intellectual disability (617125) [153]	
*CDK13*	—	PAXT kinase	Phosphorylates ZC3H14	Congenital heart defects + ID (617360) [154]	Kinase-impaired mutations in 3.9% melanoma [155]; KO mice: embryonic lethality (E16.5) with CHD, brain/kidney defects; het: CHD spectrum [156]
*LENG8*	—	PAXT TREX-2-like	Promotes decay over export	None reported	
*PCID2*	—	PAXT/TREX-2	Shared module	None reported	
*SEM1*	DSS1	PAXT/TREX-2	Shared module	Within SHFM1 locus	
**TRAMP-like Complex**					
*TENT4A*	PAPD7	Nucleolar TRAMP	Poly(A) polymerase	None reported	DC telomerase rescue; HBV target [157,158]
*TENT4B*	PAPD5	Nucleolar TRAMP	Primary DC/HBV target	None reported	PAPD5 inhibition rescues telomerase in DC [157,158]
*ZCCHC7*	AIR1	Nucleolar TRAMP	Zinc-knuckle; partners with TENT4A	None reported	Antiviral relocalization [159]
**Bridging/Adaptor Factors**					
*ARS2*	SRRT	CBCA	5′ cap sorting hub	None reported	
*NCBP1*	CBP80	CBC	Cap-binding large subunit	None reported	
*NCBP2*	CBP20	CBC	Cap-binding small subunit	3q29 deletion syndrome modifier [160]	
*ZC3H18*	—	CBCA-NEXT/PAXT	Adaptor; interacts with HP1s and Integrator	None reported	Truncating mutations in ~21% melanoma [161]
*ALYREF*	THOC4	mRNA export	Competes with ZFC3H1	None reported	
**HUSH Complex—Core**					
*MPP8*	MPHOSPH8	HUSH/HuSH2 core	Chromodomain reads H3K9me3; binds ZCCHC8	None reported	AML dependency; L1 → IFN [11,162]; KO: embryonic lethality; neural cKO: macrocephaly, Pcdh de-repression [118]
*TASOR*	FAM208A	HUSH scaffold	Pseudo-PARP; interacts with Pol II, exosome, CNOT1	None reported	HIV-1 restriction factor [97]; KO: embryonic lethality (gastrulation failure); R-loop accumulation at L1 elements [163]
*PPHLN1*	Periphilin	HUSH/HuSH2 core	RNA-binding; initiates silencing	None reported	KO: embryonic lethality (pre-E7.5)
*TASOR2*	—	HuSH2 scaffold	Targets KRAB-ZNFs and ISGs	None reported	Regulates ISGs [164]
**Chromatin-Associated Factors**					
*SETDB1*	ESET, KMT1E	HUSH effector	H3K9me3 methyltransferase	None reported	Oncogene in melanoma, lung, breast, HCC, AML; ASD [165]; KO: peri-implantation lethality; neural cKO: postnatal lethality (P10); PGC cKO: ERV de-repression; ESC deletion activates 69 ERV subfamilies
*MORC2*	—	HUSH effector	ATP-dependent chromatin compactor	CMT2Z (616688); DIGFAN (619090) [143]	L1 de-repression with DNMT1 loss [166]; S87L KI mice: neuropathy, brain apoptosis; R252W KI: peripheral neuropathies; full KO: embryonic lethality
*ATF7IP*	MCAF1	SETDB1 cofactor	Stabilizes SETDB1	None reported	
*EHMT1*	GLP, KMT1D	HUSH-associated	H3K9me1/me2 methyltransferase; feeds SETDB1 substrate	Kleefstra syndrome 1 (610253) [121,122]	Pcdh misregulation in Kleefstra [118]; Ehmt1+/− mice: Kleefstra phenocopy with cognitive/behavioral deficits, craniofacial dysmorphism [167]
*EHMT2*	G9a, KMT1C	HUSH-associated	H3K9me1/me2 methyltransferase; heterodimer with EHMT1	None reported	Overexpressed in multiple cancers
*ZNF638*	NP220	HUSH-associated	DNA-binding; recruits HUSH to retroviral DNA	None reported	Silences HBV cccDNA
*HCFC1*	HCF-1	HuSH2-associated	Transcriptional coregulator	Methylmalonic aciduria cblX (309541)	
*SIN3A*	WITKOS	HuSH2-associated	Co-repressor; HDAC complex	Witteveen-Kolk syndrome (613406)	
**HP1 Family**					
*CBX5*	HP1α	Heterochromatin	Canonical H3K9me2/3 reader; anchors exosome	None reported	Reduced in progressive MS [168]; KO mice: viable and fertile (functional redundancy with other HP1 paralogs)
*CBX1*	HP1β	Heterochromatin	Canonical H3K9me2/3 reader	None reported	Neuroinflammation; cognitive decline [169]; KO: perinatal lethality (NMJ/respiratory failure); cortical developmental defects; genomic instability
*CBX3*	HP1γ	Active gene bodies	Canonical H3K9me2/3 reader, Intronic repeats; splicing	Chromodomain deletion results in GDD, infantile muscular hypotonia, talipes valgus, strabismus, hypertrichosis and epicanthus [170]	Loss of H4K20m3, Protocadherin dysregulation [9]; Ulcerative colitis [171]; liver tumors [172] hypomorphic mice: 99% neonatal lethality; survivors: severe hypogonadism, growth restriction [173]
**Other Pathway Factors**					
*YTHDC1*	m6A reader	PAXT antagonist	nYACs shield m6A-mRNAs from exosome	None reported	Essential in AML [174]
*C9orf72*	ALS/FTD locus	Exosome substrate	Repeat RNA degraded by EXOSC10	ALS/FTD [144]	
*ATRX*	Chromatin remodeler	Heterochromatin	H3K9me3S10ph; centromeric satellite silencing	ATR-X syndrome [175]	Microglial ERV de-repression [176]; KO: embryonic lethality (E9.5); CNS cKO: postnatal lethality; neuronal cKO: spatial memory deficits; KI: ATR-X phenocopy
*SIRT6*	Chromatin/aging	LINE-1 silencing	Histone de-acetylation	None reported	L1 de-repression in aging [177,178]; KO: progeroid phenotype (death by P28); L1 de-repression drives cGAS-IFN; RT inhibition extends lifespan [178]
*LMNB1*	Lamin B1	Nuclear lamina	Loss triggers heterochromatin dissolution	None reported	Senescence biomarker; ERV reactivation [179,180]
*MB21D1*	cGAS	Innate immunity	Senses cytosolic dsDNA; inhibited by nucleosomes	None reported	Senescence/SASP; ALS; aging [181,182,183]

## 7. The Exosome in Cancer

DIS3 has emerged as a tumor suppressor in multiple myeloma. Whole-genome sequencing identified DIS3 among the most significantly mutated genes [19], with subsequent studies revealing mutations in 18.5% of newly diagnosed cases, 25% of primary plasma cell leukemia, and 30% of secondary plasma cell leukemia—predominantly missense substitutions in the RNB catalytic domain [20]. Germline DIS3 loss-of-function variant segregating in familial myeloma kindreds established DIS3 as a hereditary susceptibility gene [21].

Cancer cells often appear to evade exosome surveillance without mutating exosome components directly. In acute myeloid leukemia (AML), the m^6^A reader YTHDC1 forms nuclear condensates that phase-separate m^6^A-modified mRNAs away from the PAXT complex and exosome, shielding leukemogenic transcripts; dissolution of these condensates restores exosome-mediated degradation and triggers differentiation [174]. A complementary kinetic escape may often operate via gene gating: rapid co-transcriptional export of oncogenic transcripts such as MYC may limit nuclear residence time below the threshold for exosome interrogation [184]. The same evasion logic scales beyond the malignant cell itself. A growing catalog of oncogenic and immunomodulatory long noncoding RNAs such as MALAT1, NEAT1, HOTAIR, and lnc-TIM3 are stable, functional transcripts that reprogram the tumor microenvironment, toggling immune cells between antitumor effector and immunosuppressive states [185]. Because such lncRNAs must first accumulate to act, they represent a phenotypic endpoint of transcripts that have escaped nuclear RNA exosome surveillance: whether through condensate sequestration, kinetic export, or simply intrinsic stability, transcripts that evade degradation are precisely those free to acquire regulatory function. This reframes exosome surveillance not only as a brake on cell-intrinsic oncogenesis but as an upstream constraint on the noncoding regulatory repertoire available to remodel the tumor immune landscape.

Oncogenic CDK13 mutations (3.9% of cutaneous melanomas) disrupt PAXT by failing to phosphorylate ZC3H14 [153], thereby stabilizing prematurely terminated RNAs whose expression alone drives melanomagenesis in zebrafish [155]. Systematic analysis is defining a substrate code for PAXT target recognition [63], and recurrent mutations in additional PAXT components across cancer types, including truncating ZC3H18 mutations, with up to 21% of melanomas carrying nuclear RNA surveillance pathway mutations [161], establish a broad tumor-suppressive role for nuclear RNA surveillance. Germline CDK13 mutations cause congenital heart defects, dysmorphic features, and intellectual disability, placing CDK13 at the intersection of developmental and oncogenic pathology through its regulation of nuclear RNA surveillance.

## 8. Age-Related Changes in Exosome Function Lead to Transposable Element De-Repression and Cellular Senescence

**Figure 4:**
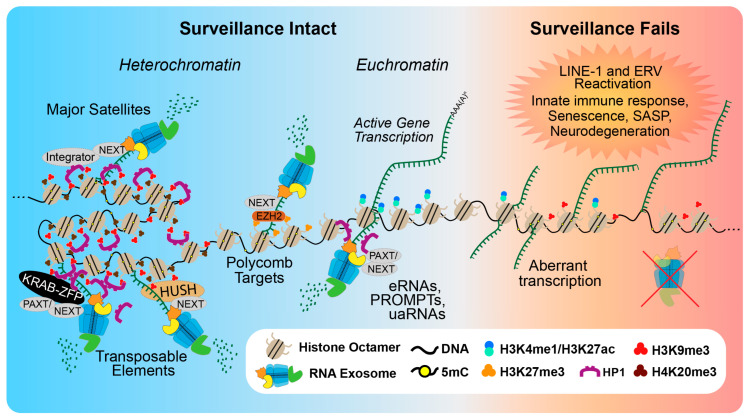
The nuclear RNA exosome couples RNA decay to chromatin state, and the consequences of decoupling. A single chromatin fiber is shown decompacting from left to right, from constitutive heterochromatin through Polycomb chromatin and active euchromatin to a state of surveillance failure. Left (surveillance intact): Each chromatin state delivers its nascent transcripts to the same exosome by a state-specific route. At pericentric major satellites, cap-less, poly(A)-deficient transcripts are terminated by Integrator and routed through NEXT. At transposable elements, sequence-specific KRAB-ZFP-KAP1 repression and RNA-feature-based HUSH repression converge on H3K9me3/HP1; full-length, polyadenylated elements are routed through PAXT and shorter, poly(A)-deficient transcripts through NEXT. At Polycomb targets, nascent lncRNA turnover by NEXT sets the pool of EZH2/PRC2 available to chromatin. At active genes and enhancers, eRNAs, PROMPTs and uaRNAs are cleared by NEXT and PAXT, tuning regulatory-RNA levels without depositing a repressive mark. Right (surveillance fails): Loss of exosome activity (crossed-out complex) stabilizes these transcripts, heterochromatin decompacts with loss of H3K9me3 and DNA methylation, and aberrant transcription and LINE-1/ERV reactivation ensue—driving innate immune activation, senescence and SASP, and neurodegeneration. Histone marks, HP1, 5mC and the exosome are keyed in the inset legend.

Age-related erosion of the heterochromatin-RNA surveillance axis permits transposable element de-repression, linking exosome decline to senescence and neurodegeneration. Approximately 45% of the human genome derives from transposable elements [186]. Their silencing operates through layered defense: transcriptional repression via KRAB zinc finger proteins recruiting KAP1 and SETDB1 to ERVs [97,98], KRAB zinc finger proteins [91], and DNA methylation as the primary silencing mechanism, with HUSH-MORC2 providing a secondary layer [166], followed by post-transcriptional surveillance via the NEXT-exosome axis at the same loci [8]. This dual architecture ensures that transcripts escaping incomplete silencing are captured before reverse transcription or innate immune activation. This layered architecture and the consequences of its erosion are summarized in Figure 4.

Aging is accompanied by progressive erosion of the heterochromatin barriers that restrain transposable elements, with LINE-1 retrotransposons serving as both a marker and a driver of age-related inflammation. LINE-1 retrotransposons become progressively de-repressed during aging as SIRT6 is depleted from L1 loci [177]. In aged mice, LINE-1 de-repression activates cGAS-STING via reverse-transcribed cytoplasmic cDNA, triggering type I interferon responses rescuable by reverse-transcriptase inhibitors [178]. Nanopore sequencing has confirmed that somatic LINE-1 insertions accumulate in the aging human brain, with decreased CpG methylation at young L1 elements in Alzheimer’s disease [187]. Loss of B-type lamins leads to heterochromatin dissolution and ERV reactivation; multi-omics profiling showed that abacavir, another nucleoside reverse-transcriptase inhibitor, alleviates these phenotypes [180].

Transposable element de-repression has emerged as a convergent pathological mechanism across neurodegenerative diseases, linking heterochromatin erosion to neuroinflammation. Postmitotic neurons cannot dilute accumulated damage through cell division and must therefore maintain surveillance for decades. It follows that even modest declines in exosome activity or epigenetic repression, compounded over a lifetime, permit gradual accumulation of retrotransposon-derived transcripts that eventually breach the cGAS-STING activation threshold. In a fly model of Alzheimer’s disease, tau promotes heterochromatin loss, permitting aberrant TE transcription, with genetic rescue of heterochromatin integrity reducing neurodegeneration in Drosophila [188]. Recent evidence suggests that in mammals, tau can enter the nucleus and perturb laminar heterochromatin and interact with HP1γ [189]. Given TE products can themselves promote protein aggregation and induce the integrated stress response, this can establish a feed-forward loop [190]. A conditional HP1β/γ knockout in cortical neurons modeled accelerated brain aging, where progressive ERVK/IAP de-repression coincided with stimulation of complement C3-positive reactive astrocytes and phagocytic microglia, cell types seen during normal aging. This environment sees age-dependent reductions in dendritic complexity and cognitive decline [9]. These converging lines of evidence establish TE de-repression as a unifying mechanism across neurodegenerative diseases [191].

Cellular senescence provides the most direct evidence that exosome decline is a causal driver, rather than a mere correlate, of the inflammatory phenotypes associated with transposable element de-repression. Senescent cells exhibit reduced RNA turnover driven by decreased exosome subunit expression [192]. The resulting accumulation of retrotransposon-enriched promoter-associated RNAs generates dsRNA that activates interferon signaling and the senescence-associated secretory phenotype (SASP). EXOSC3 depletion alone is sufficient to accelerate senescence, establishing exosome decline as a causal factor rather than a correlate. In ESCs, acute EXOSC2 depletion triggers rapid nuclear RNA aggregation, impaired Pol II initiation, translational shutdown, and senescence-like chromatin changes [22]. Senescent cells undergo progressive lamin B1 loss [179], heterochromatic foci dissolution, and TE de-repression [22,192]. In neurons this senescence is further amplified by postmitotic programs [193]. Senescent cells also release chromatin-laden extracellular particles that activate cGAS-STING in recipient cells, potentially propagating inflammation beyond the senescent cell [194]. Cytosolic chromatin fragments from lamin B1 degradation can activate cGAS, and this signaling is required for full SASP elaboration [179,181]. Intact nucleosomal DNA is a poor cGAS activator, whereas partially histone-stripped fragments are potent activators [183].

## 9. Pharmacological Interventions

The mechanisms outlined above suggest three distinct points of therapeutic entry: suppressing the downstream consequences of surveillance failure, manipulating the enzymes that mark transcripts for exosomal decay, and restoring surveillance capacity itself. Clinical progress to date is confined almost entirely to the first.

The first entry point accounts for most clinical activity to date, and the cascade it targets can be intercepted at successive stages. Reverse-transcriptase inhibitors, repurposed from antiretroviral therapy, act on the retrotransposon-driven inflammation of aging and senescence rather than on the exosome, and the rationale for their use is preclinical [178,180]. Human evidence remains early: in a 24-week open-label phase IIa trial in twelve participants with predementia Alzheimer’s disease, the nucleoside analog lamivudine met its primary endpoints of safety, tolerability and feasibility, and among exploratory biomarkers lowered cerebrospinal fluid (CSF) glial fibrillary acidic protein, a marker of astrocytic activation, and raised the plasma Aβ42/40 ratio [195]. The trial was uncontrolled, and reverse-transcriptase activity was unchanged in plasma and undetectable in CSF, so central target engagement remains to be demonstrated. Further along the same cascade, senolytics remove the senescent cells that sustain the secretory phenotype rather than the transcripts that provoke it: the senolytic combination dasatinib plus quercetin has entered open-label testing in early Alzheimer’s disease, with reported outcomes so far limited to CSF drug penetration, feasibility and safety [196]. Even further downstream at the sensing step, pharmacological STING inhibition in aged mice suppressed inflammatory signatures and improved cognition, with microglial cGAS engagement sufficient to drive bystander neuroinflammation [197]. All three suppress the consequences of surveillance failure without restoring surveillance itself.

The second point of entry lies closer to the machinery. PAPD5 (TENT4B) and PAPD7 (TENT4A) are the non-canonical poly(A) polymerases of the mammalian TRAMP complex introduced in Section 3; they append oligo(A) tails to RNA 3′ ends, tagging transcripts for nuclear exosome degradation. This activity is therapeutically double-edged: because PAPD5 oligoadenylation destabilizes the telomerase RNA component TERC, small-molecule PAPD5/7 inhibitors such as RG7834 raise TERC levels, restore telomerase activity, and rescue the hematopoietic defects of dyskeratosis congenita [158], whereas the same enzymes instead stabilize hepatitis B virus RNA, so their inhibition accelerates viral RNA decay and suppresses HBV [157]. That a single class of inhibitor is beneficial in two unrelated diseases illustrates how directly the exosome-targeting polyadenylation step can be drugged, positioning the RNA-surveillance machinery itself as an emerging therapeutic axis.

Interventions to date therefore address either the downstream consequences of surveillance failure or the enzymes that tag transcripts for decay. The third entry point—restoring surveillance capacity itself—has yet to reach the clinic, but it is not without precedent. Pan-neuronal expression of human EXOSC3 rescues neurodegeneration in a Drosophila PCH1b model [136], and in acute myeloid leukemia, dissolving the YTHDC1 condensates that sequester transcripts away from PAXT reinstates exosome-mediated degradation and triggers differentiation [174]. Restoring access to an exosome that is already present may prove more tractable than augmenting the complex itself.

## 10. Future Directions

We have argued that the nuclear RNA exosome functions not as a disposal system but as a guardian of the epigenome. From yeast to humans, the exosome and its adaptors degrade noncoding and TE-derived transcripts as an active mechanism of epigenetic enforcement: shaping chromatin states through HUSH and HP1, constraining enhancer activity, preserving 3D genome architecture, and limiting R-loop and retrotransposon accumulation. When surveillance fails, the consequences extend from local chromatin decompaction to global innate immune activation (Figure 4).

Several key questions remain. First, how is exosome activity regulated during development? The “adaptor fingerprint” concept provides a framework, but systematic single-cell profiling of adaptor expression across human tissues has yet to be performed. Second, what determines tissue-specific vulnerability in exosomopathies? The cell-type-resolved atlas of Higginson et al. [136] has begun to address this question, demonstrating that Kenyon cells (mushroom body neurons) are particularly vulnerable in Drosophila, paralleling cerebellar vulnerability in human PCH. Third, how can exosome activity be best therapeutically modulated? CDK13 mutations disrupting PAXT suggest that restoring nuclear RNA degradation could have antitumor effects. Reverse-transcriptase inhibitors alleviating aging phenotypes [178,180] have prompted clinical investigation in neurodegeneration [195]. Finally, is exosome decline a druggable driver of aging? EXOSC3 depletion triggers senescence [192], suggesting that maintaining exosome expression could delay SASP onset, although achieving this without disrupting surveillance selectivity remains an open challenge.

## Data Availability

No new data were created or analyzed in this study. Data sharing is not applicable to this article.

## Abbreviations

The following abbreviations are used in this manuscript:

AD	Alzheimer’s Disease
AID	Activation-Induced Cytidine Deaminase
ALS	Amyotrophic Lateral Sclerosis
AML	Acute Myeloid Leukemia
ARE	AU-Rich Element
ARS2	Arsenite Resistance Protein 2 (SRRT)
ATF7IP	Activating Transcription Factor 7-Interacting Protein
ATRX	Alpha Thalassemia/Mental Retardation Syndrome X-Linked
C1D	C1D Nuclear Receptor Co-repressor (Rrp47)
CBCA	Cap-Binding Complex-ARS2
CBX1	Chromobox Protein Homolog 1 (HP1β)
CBX3	Chromobox Protein Homolog 3 (HP1γ)
CBX5	Chromobox Protein Homolog 5 (HP1α)
CDK13	Cyclin-Dependent Kinase 13
cenRNA	Centromeric RNA
cGAMP	Cyclic GMP-AMP
cGAS	Cyclic GMP-AMP Synthase
ChIP-seq	Chromatin Immunoprecipitation Sequencing
CMT	Charcot-Marie-Tooth Disease
cPcdh	Clustered Protocadherin
CRISPR	Clustered Regularly Interspaced Short Palindromic Repeats
cryo-EM	Cryogenic Electron Microscopy
CSR	Class-Switch Recombination
CTCF	CCCTC-Binding Factor
CUT	Cryptic Unstable Transcript
daRNA	Downstream Antisense RNA
DIS3	Dis3 Homolog, Exosome Endoribonuclease and 3′→5′ Exoribonuclease
DIS3L	DIS3-Like Exoribonuclease 1
DIS3L2	DIS3-Like Exoribonuclease 2
DNMT	DNA Methyltransferase
dsRNA	Double-Stranded RNA
EHMT1	Euchromatic Histone Lysine Methyltransferase 1 (GLP)
eRNA	Enhancer RNA
ERV	Endogenous Retrovirus
ESC	Embryonic Stem Cell
Exo-9	Nine-Subunit Exosome Core
FTD	Frontotemporal Dementia
FTLD	Frontotemporal Lobar Degeneration
H3K27me3	Histone H3 Lysine 27 Trimethylation
H3K4me3	Histone H3 Lysine 4 Trimethylation
H3K9me2	Histone H3 Lysine 9 Dimethylation
H3K9me3	Histone H3 Lysine 9 Trimethylation
HCC	Hepatocellular Carcinoma
HERVL	Human Endogenous Retrovirus L
HP1	Heterochromatin Protein 1
HUSH	Human Silencing Hub
IAP	Intracisternal A-Particle
IBD	Inflammatory Bowel Disease
IFN	Interferon
iPSC	Induced Pluripotent Stem Cell
IRF2	Interferon Regulatory Factor 2
ISG	Interferon-Stimulated Gene
KAP1	KRAB-Associated Protein 1 (TRIM28)
KRAB-ZFP	Krüppel-Associated Box Zinc Finger Protein
LINE-1	Long Interspersed Nuclear Element 1
lncRNA	Long Noncoding RNA
LTR	Long Terminal Repeat
m6A	N6-Methyladenosine
MAVS	Mitochondrial Antiviral-Signaling Protein
MDA5	Melanoma Differentiation-Associated Protein 5
MERVL	Mouse Endogenous Retrovirus L
MiCEE	Mirlet7d-C1D-Exosome-EXOSC10 Complex
MM	Multiple Myeloma
MORC2	MORC Family CW-Type Zinc Finger 2
MPP6	M-Phase Phosphoprotein 6
MPP8	M-Phase Phosphoprotein 8 (MPHOSPH8)
mRNA	Messenger RNA
MTR4	mRNA Transport 4 (DExH-box RNA Helicase)
ncRNA	Noncoding RNA
NEXT	Nuclear Exosome-Targeting Complex
OPMD	Oculopharyngeal Muscular Dystrophy
PABPC1	Poly(A)-Binding Protein Cytoplasmic 1
PABPN1	Poly(A)-Binding Protein Nuclear 1
PAXT	Poly(A) Tail Exosome-Targeting Connection
Pcdh	Protocadherin
PCH	Pontocerebellar Hypoplasia
Pol II	RNA Polymerase II
PRC2	Polycomb Repressive Complex 2
PROMPT	Promoter Upstream Transcript
R-loop	RNA:DNA Hybrid with Displaced Single-Stranded DNA
RAN	Repeat-Associated Non-AUG Translation
RBM7	RNA-Binding Motif Protein 7
RIG-I	Retinoic Acid-Inducible Gene I
rRNA	Ribosomal RNA
SASP	Senescence-Associated Secretory Phenotype
SETDB1	SET Domain Bifurcated Histone Lysine Methyltransferase 1
SHM	Somatic Hypermutation
SHRF	Short Stature, Hearing Loss, Retinitis Pigmentosa, and Distinctive Facies
SKI	Superkiller Complex
SKIV2L	Superkiller Viralicidic Activity 2-Like
SMA	Spinal Muscular Atrophy
snoRNA	Small Nucleolar RNA
snRNA	Small Nuclear RNA
STING	Stimulator of Interferon Genes
SVA	SINE-VNTR-Alu Retrotransposon
TAD	Topologically Associating Domain
TASOR	Transgene Activation Suppressor (FAM208A)
TASOR2	TASOR Paralog (FAM208B)
TE	Transposable Element
TERC	Telomerase RNA Component
THES	Trichohepatoenteric Syndrome
TRAMP	Trf4/5-Air1/2-Mtr4 Polyadenylation Complex
TREX1	Three Prime Repair Exonuclease 1
TRIM28	Tripartite Motif-Containing 28
TTC37	Tetratricopeptide Repeat Domain 37 (SKI3/Thespin)
WAPL	Wings Apart-Like Protein Homolog
YTHDC1	YTH Domain-Containing Protein 1
ZC3H18	Zinc Finger CCCH-Type Containing 18
ZCCHC8	Zinc Finger CCHC-Type Containing 8
ZFC3H1	Zinc Finger C3H1-Type Containing
